# No evidence of spontaneous preference for slowly moving objects in visually naïve chicks

**DOI:** 10.1038/s41598-020-63428-3

**Published:** 2020-04-14

**Authors:** Bastien S. Lemaire

**Affiliations:** Center for Mind/Brain Sciences, University of Trento, Piazza Manifattura, 1, 38068 Rovereto, TN Italy

**Keywords:** Psychology, Neuroscience

## Abstract

It has been recently reported that young chicks that have received equal exposure to slowly- and fast-rotating objects showed a preference for slowly-rotating objects. This would suggest that visual experience with slowly moving objects is necessary for object recognition in newborns. I attempted to duplicate this finding in newborn chicks using a simple rotating blue cube. No significant preference was found. Using objects similar to the ones used in the previous study (digital embryos), I observed a strong and robust preference for the fast- (not for the slow-) rotating object. To clarify whether the discrepancies with the previous study could be due to the stimuli frame-frequency used (the chicks’ visual system is characterized by high temporal resolution), I repeated the experiments by presenting the stimuli with a lower-frame frequency (from 120 fps to 24 fps). However, similar preferences for the fast-rotating objects were found, this time also for the rotating blue cube. These results suggest a preference for fast-rotating objects that is modulated by the shape and, in part, by the frame-frequency. It remains to be established whether the discrepancies between this study and the previous study can be explained by differences related to strains or artefacts due to the use of monitors with a low-refresh rate.

## Introduction

The domestic chick is one of the most used avian models in laboratories. One of its advantages lies in its relative maturity and mobility at ‘birth’^1^, which allows researchers to disentangle learned from unlearned knowledge. Over the past years, numerous studies have shed light on the cognitive and perceptual abilities possessed by naïve birds^2,3^. With limited and controlled experience, chicks show arithmetic capacities^4–6^ and are able to use multiple information for object individuation^7,8^. Furthermore, like human newborns, they demonstrate intuitive physics^9–11^ and perceive the visual Ebbinghaus illusion^12^. To demonstrate these amazing capacities, researchers have been taking advantage of a specific phenomenon called filial imprinting.

Filial imprinting has been described as a learning process through which the social behaviour of a young becomes restricted to a particular individual or object^13–19^. Precocial animals such as domestic chicks develop a strong preference toward a stimulus in a short period of time. In this context, chicks have to find the right kind of stimuli quickly to imprint on and survive.

Decades of research on filial imprinting and the domestic chick have led to the discovery of many predispositions that help animals to orient toward specific stimuli features (sizes^20,21^, shapes^22^, colours^23–26^). Furthermore, the predispositions described in precocial species such as the domestic chick are not only species-specific. For example, both human newborns and naïve domestic chicks have a preference for face-like stimuli^27–33^. This suggests shared mechanisms and demonstrates the importance in studying precocial species in a comparative perspective^1,34^.

Specific patterns of motion are more likely to be perceived as animate in adults^35^ and newborns humans^36–38^. In line with these findings, several studies in chicks have described spontaneous preferences for patterns that drive animacy detection. Chicks have a preference for biological motion (semi-rigid movement representing animal’s motion) in comparison to rigid and random motion^39–41^. Furthermore, they prefer objects that can accelerate and decelerate (self-propelled) in comparison to objects moving at a constant speed^42^. It has also been found that chicks have a preference for objects that rotate and move along their main body axis^43,44^.

Interestingly, a recent study^45^ reported a novel spontaneous motion preference in chicks. In this study, a spontaneous and growing preference for slow- (in comparison to fast-) moving objects was described. The author interpreted this finding based on the idea that visual experience with slowly moving objects would help animals to build accurate object representation, whereas exposure to quickly moving objects would create inaccurate object representation.

Motion has been described as an essential cue to attract chicks’ attention since imprinting’s early work^14,15,20^. However, in this context, it seems that the faster an object moves, the more attractive it becomes^46^. James^47–49^ demonstrated that chicks were more attracted by an object located close to a fast- (in comparison to slow-) flickering light. Fast-flickering light was thus more attractive and led the animals to spend more time with the object close to it.

Based on the filial imprinting literature, the preference observed for slow-moving objects is thus entirely unexpected, and for this reason theoretically fascinating. Consequently, I decided to replicate the study using a similar method to re-investigate whether newly-hatched chicks need visual experience with slowly moving objects to perceive the world successfully. Three experiments were performed by manipulating the shape and the frame frequency of the stimuli.

## Experiment 1

### Methods

The study was carried out in compliance with the European Union and the Italian law on the treatment of animals. The experimental procedures were approved by the Ethical Committee of the University of Trento and licenced by the Italian Health Ministry (permit number 630/2018).

The methods used were similar to the first experiment of the study of Wood^45^. Soon after hatching, visually naïve chicks were placed in a cage for five days and stimulated with two versions of a stimulus, a slow- and fast-rotating stimulus. In this experiment, I tested whether chicks preferred a slow- or a fast-rotating cube.

#### Subjects

Ten chicks (seven females) domestic chicks (*Gallus gallus*) of the strain Ross 308 were used. The sample size was determined based on Wood’s previous studies^50,51^ using automated controlled rearing experiments. The eggs were sourced from a commercial hatchery (Azienda Agricola Crescenti) and were incubated in the laboratory under controlled conditions (37.7 °C and 40% of humidity). Three days before hatching, chicks were transferred into opaque individual boxes and placed inside a hatching chamber at 37.7 °C and 60% of humidity. All operations were performed in darkness to prevent the chicks from having visual experience before the start of the experiment.

#### Apparatus

**Figure 1:**
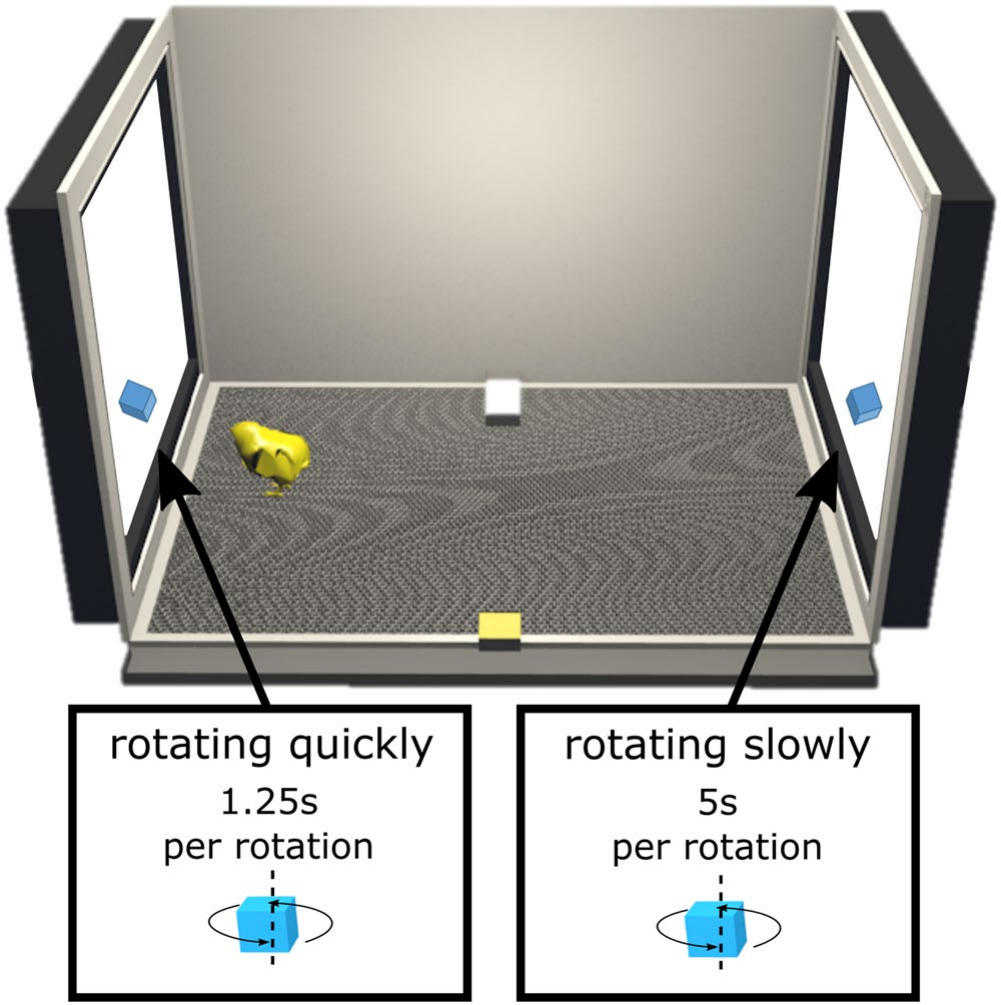
Schematic representation of the apparatus and stimuli used in the first experiment. The position of the stimuli on the screens was switched and balanced across sessions.

Multiple apparatuses were used at the same time to test several animals simultaneously. Each apparatus had a rectangular shape (90 cm × 60 cm ×60 cm, Fig. 1). A high-frequency screen (ASUS MG248QR, 120 Hz) was located on each smaller wall of the apparatus to display stimuli. Animal’s behaviour was recorded using a Microsoft life camera placed 105 cm above the ground. Food and water were located in the middle of each larger wall and available ad libitum.

#### Stimuli

The stimulus used was a 3-dimensional tilted cube (5 cm) coloured in blue (see Fig. 1 and video in the supplementary materials). Two versions of the stimulus were created. On the first version, the blue cube was rotating on itself quickly (one rotation every 1.25 s). On the second version, the same cube was rotating on itself slowly (one rotation every 5 s). The use of these rotatory speeds has been described to produce a preference for slow motion in Wood’s second experiment^45^. The videos were exported with a high frame rate (120 frames per second) to ensure a smooth displaying of the stimuli. The stimuli were created and animated using Blender (version 2.79). The screen frequency was set at 120 Hz to avoid flicker.

#### Procedure

After hatching chicks were sexed (using night-vision goggles) and singly placed in their apparatus for five days in a day-night cycle (LD 13:11 hr). Sexing was necessary because the difference in the direction of responses of male and female chicks have been reported in several laboratories^39,52–54^. During the day, the chicks underwent 13 sessions of 59 minutes of test with the stimuli displayed on the screens (fast-moving stimulus displayed on one screen and slow-moving stimulus on the other). Between sessions, the displaying of the stimuli was interrupted by 1 minute of dark screens. The position of the stimuli on the screens was balanced across sessions. During the night, dark screens were displayed.

#### Data analysis

The position of the animal (centre of the body) within the cage was scored automatically using DeepLabCut, an open-source deep-learning toolbox made to efficiently track animal behaviours^55^. The preference for a stimulus was assessed using the time spent close to it. Consequently, the apparatus had been divided into three different zones. Two screen zones (30 cm by 60 cm) and a centre zone (30 cm by 60 cm). A preference score had been calculated using the following

$${\rm{formula}}:\,\frac{{\rm{time}}\,{\rm{spent}}\,{\rm{by}}\,{\rm{the}}\,{\rm{slow}}-{\rm{moving}}\,{\rm{stimulus}}}{{\rm{time}}\,{\rm{spent}}\,{\rm{by}}\,{\rm{the}}\,{\rm{two}}\,{\rm{stimuli}}}\times 100$$

Using this formula, a score of 50% indicates no preference for either stimulus. A score higher than 50% indicates a preference for the slow-rotating stimulus, while a score lower than 50% indicates a preference for the fast-rotating stimulus.

To determine whether chicks’ preferences were influenced by sex and testing day, I performed a mixed ANOVA. The distribution of the residuals was checked (Q-Q plots) and validated as normally-distributed. Sphericity was checked using Mauchly’s test, and corrections were performed when the assumptions were not met.

The preference toward a stimulus (different from chance-level) was evaluated using two-tailed one-sample t-tests and also by estimating the relative likelihood ratio or Bayes factor (BF_10_). The latest allows to demonstrate how likely the overall preference was different from the null hypothesis (H0).

Statistical analyses were performed using RStudio^56^ and the following packages: *ez*^57^, *goftest*^58^, *nlme*^59^, *lme*^60^, *tidyr*^61^, *plyr*^62^, *dplyr*^63^, *reshape*^64^, *lsr*^65^*, BayesFactor*^66^ and *ggplot*2^67^.

### Results

**Figure 2:**
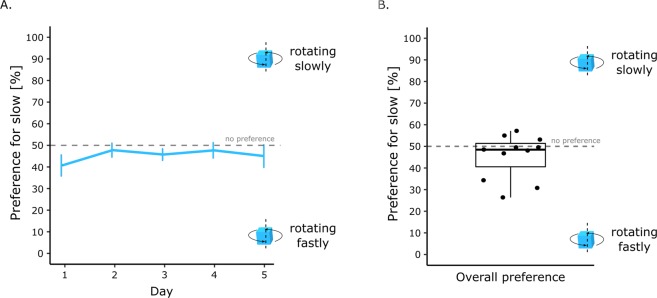
(**A**) Preference score between the slow- and fast-rotating cube across testing days. (**B**) Overall preference score between the slow- and fast-rotating cube.

The results are shown in Fig. 2. There were no significant effects associated with sex (*F*(1, 9) = 0.17, *p* = 0.90, *η*^2^_*G*_ = 0.001), day (*F*(4, 36) = 1.12, *p* = 0.36, *η*^2^_*G*_ = 0.044) or interaction (sex x day, *F*(4, 36) = 1.37, *p* = 0.26, *η*^2^_*G*_ = 0.054). The preference score was not significantly different from chance level (*t*(10) = −1.50, *p* = 0.16, Cohen’s *d* = 0.45). There was however a trend for a preference for the fast-rotating object. Chicks spent in average 55% (+/− 3.08 SEM) of their time close the fast-rotating cube (Fig. 2B).

Furthermore, the BF_10_ for the overall preference, which is 0.72, confirmed that the preference observed is not different from the null hypothesis.

## Experiment 2

The results of the first experiment did not reveal any clear preference. This could be due to the shape of the stimulus used. For example, It has been reported that, at least in human vision, the number of corners and contour structure can affect motion perception of rotatory objects^68^. Besides, the colour of the object might influence the approach behaviours of the animals towards the stimuli. Several imprinting studies demonstrated that some colours are more attractive than others in domestic chicks^19^. Red and blue have been described to be equally attractive in comparison to green and yellow^24–26^. It has also been described that chick’s favourite colour is influenced by experience and genetic differences^23^. Therefore, I conducted a second experiment by using another type of stimuli similar in shape and colour to those used in the study of Wood^45^.

### Methods

**Figure 3:**
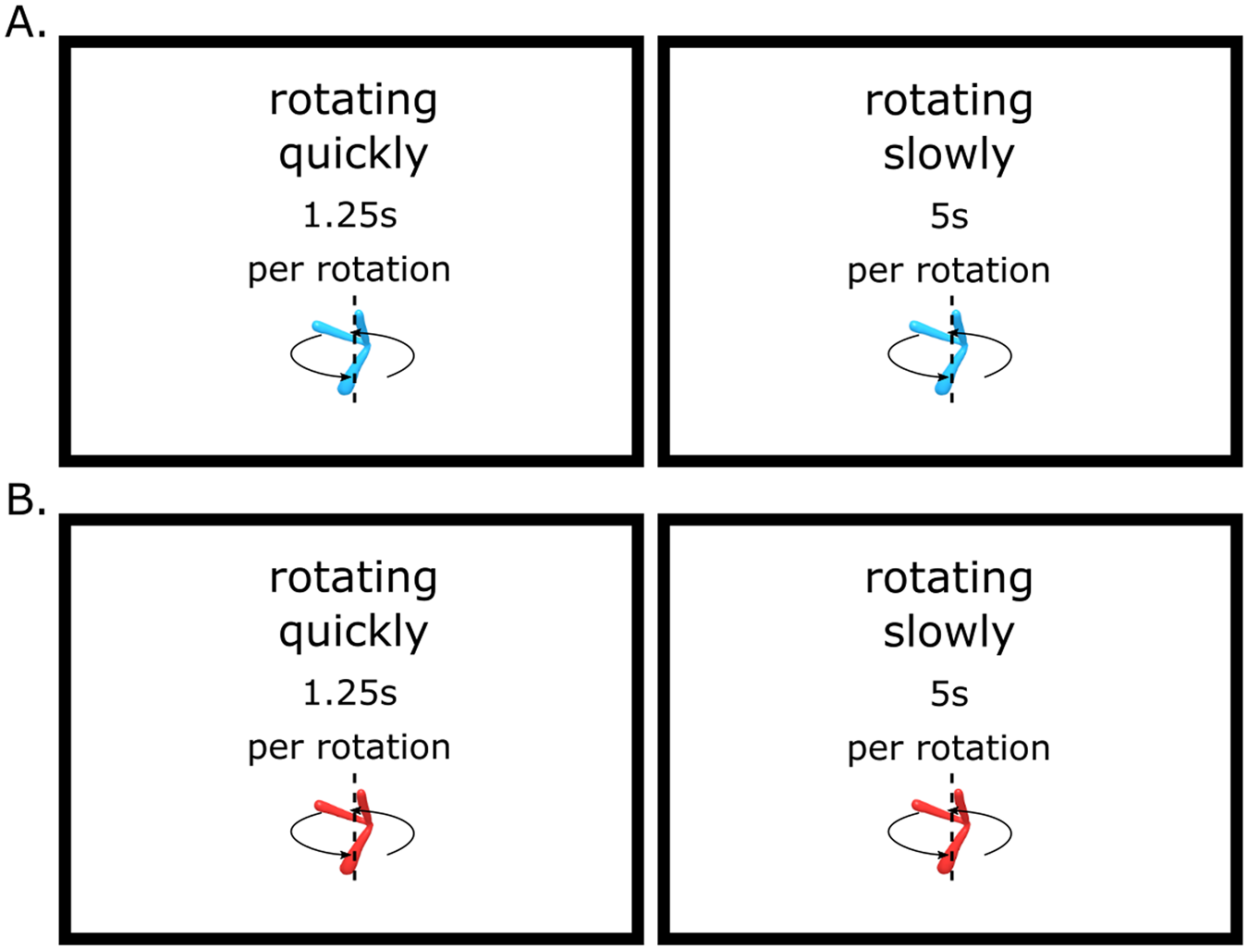
Representation of the digital embryos in blue (**A**) and red (**B**).

The methods were the same as in the first experiment except for the stimulus used. The new stimulus (Digital Embryo) had been generated using a program called Digital Embryo Workshop (DEW). The stimulus was then imported, animated and exported in two versions (red and blue, see Fig. 3 and video in the supplementary materials) using Blender (v2.79).

Ten chicks (four females) were tested using the red digital embryo. Eleven chicks (four females) were tested using the blue digital embryo (Fig. 3).

### Results

**Figure 4:**
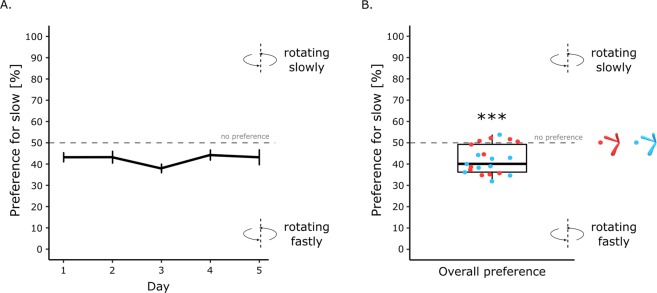
(**A**) Preference score between the slow- and fast-rotating Digital Embryos (blue and red) across testing days. (**B**) Overall preference score between the slow- and fast-rotating Digital Embryos (p < 0.001, ***). The red dots represent the overall individual preference of the chicks tested with the red Digital Embryo. The blue dots represent the overall individual preference of the chicks tested with the blue Digital Embryo.

The results are shown in Fig. 4. There were no significant effects associated with sex (*F*(1, 17) = 0.18, *p* = 0.89, *η*^2^_*G*_ = 0.0004), colour (*F*(1, 17) = 0.017, *p* = 0.25, *η*^2^_*G*_ = 0.027), day *(F*(4, 68) = 1.10, *p* = 0.36, *η*^2^_*G*_ = 0.042) or interactions (sex x colour, *F*(1, 17) = 0.43, *p* = 0.52, *η*^2^_*G*_ = 0.0083; sex x day, *F*(4,68) = 0.26, *p* = 0.90, *η*^2^_*G*_ = 0.010; colour x day, *F*(4, 68) = 0.23, *p* = 0.92, *η*^2^_*G*_ = 0.0090; sex x colour x day, *F*(4, 68) = 0.28, *p* = 0.89, *η*^2^_*G*_ = 0.011). The preference score was significantly different from chance level (*t*(20) = −5.26, *p* < 0.001, Cohen’s *d* = 1.15). The majority of the chicks chose the fast-rotating object and spent in average 58% (+/− 1.50 SEM) of their time close to it (Fig. 4).

Furthermore, the BF_10_ revealed that the overall preference score is 653 times more likely than the null hypothesis. Following Lee and Wagenmakers classification^69^, the BF_10_ provides extreme evidence that the chicks possess a strong preference for the fast-rotating digital embryos.

## Experiment 3

This experiment confirmed that the shape of an object likely influences the perception of rotatory motion (though a similar trend was also observed in Exp. 1). Nonetheless, the preference observed is opposite to that reported in Wood’s study^45^. One reason for this discrepancy could be associated with the frame frequency of the videos displaying the stimuli. In the replicated study^45^, videos with a low frame rate (24 fps) were used, whereas videos with a high frame rate (120 fps) were used in this study. Birds possess highly developed visual systems which make them perceive the environment in slow motion in comparison to what humans perceive^70^. In chicks, the temporal perception (quantified by using critical flicker fusion, CFF) is higher than in humans and can reach 115 Hz in some individuals^71,72^. It is thus possible that the videos displayed at 24 fps were perceived as a series of still images rather than a smooth motion by the young birds, thereby altering their motion perception.

To test this hypothesis, in the third experiment I used the same stimuli employed in the first and second experiment but changed their frame rate (fps) from a high to low fps value (such as that used in Wood^45^).

### Methods

Similar methods were used than in previous experiments except for the frame rate of the displaying of the stimuli, which were decreased from 120 to 24 fps using Blender.

Twelve chicks (six females) were tested using the blue cube. Eleven chicks (five females) were tested using the red Digital Embryo. Eleven chicks (five females) were tested using the blue Digital Embryo.

### Results

**Figure 5:**
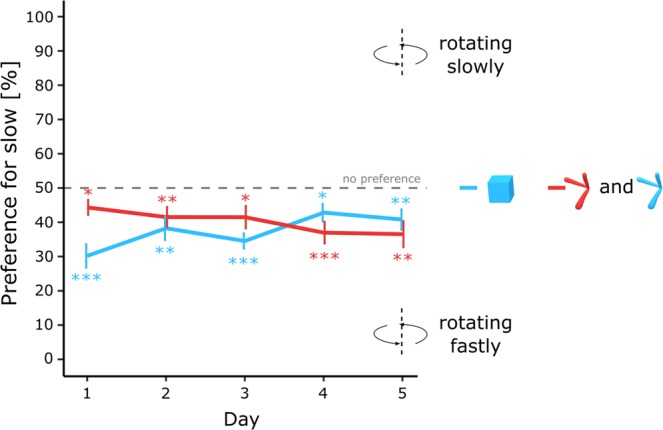
Preference score between a slow- and fast-rotating objects (cube and Digital Embryos) across testing days (p < 0.05, *, p < 0.01, **, p < 0.001, ***). The blue line represents to the preference score of the chick stimulated by the blue cube whereas the red line represents the preference score of the chicks stimulated by the Digital Embryos (blue and red Digital Embryos pulled together).

The results are shown in Fig. 5. First, I checked for any effect of colour within the group of chicks exposed to the Digital Embryos. There were no significant effects associated with colour (*F*(1, 18) = 1.04, *p* = 0.32, *η*^2^_*G*_ = 0.035), sex (*F*(1, 30) = 0.13, *p* = 0.72, *η*^2^_*G*_ = 0.050) or interactions (sex x colour, *F*(1, 18) = 1.05, *p* = 0.32, *η*^2^_*G*_ = 0.035; colour x day, *F*(4, 72) = 0.34, *p* = 0.20, *η*^2^_*G*_ = 0.0072; sex x colour x day, *F*(4, 72) = 0.45, *p* = 0.77, *η*^2^_*G*_ = 0.0094) on the preference score.

Since, they were no effect of colour, chicks exposed to the blue and red Digital Embryos were grouped together for the subsequent analyses. There were no significant effects associated with stimulus (*F*(1, 30) = 0.51, *p* = 0.48, *η*^2^_*G*_ = 0.0098), sex (*F*(1, 30) = 0.13, *p* = 0.72, *η*^2^_*G*_ = 0.0025) or interactions between sex and stimulus (*F*(1, 30) = 1.40, *p* = 0.20, *η*^2^_*G*_ = 0.032).

Mauchly’s test indicated that the assumption of sphericity had been violated for one factor (day, *W* = 0.46, *p* < 0.01) and several interactions (day x sex, *W* = 0.46, *p* < 0.01; day x stimulus, *W* = 0.46, *p* < 0.01; day x sex x stimulus, *W* = 0.46, *p* < 0.01). Therefore, degrees of freedom were corrected using Huynh-Feldt estimates of sphericity. There was a significant interaction between testing day and stimulus (*F*(1.82, 54.66) = 6.07, *p* < 0.001, *η*^2^_*G*_ = 0.078) but no significant effect of day (*F*(1.82, 54.66) = 0.44, *p* = 0.76, *η*^2^_*G*_ = 0.0060) or other interactions (day x sex, *F*(1.82, 54.66) = 2.13, *p* = 0.10, *η*^2^_*G*_ = 0.029; day x sex x stimulus, *F*(1.82, 54.66) = 0.10, *p* = 0.97, *η*^*2*^_*G*_ = 0.0014).

The preference scores were significantly different from chance-level on each testing day with the cube (day 1, *t*(11) = −5.82, *p* < 0.001, Cohen’s *d* = 1.68, day 2, *t*(11) = −3.45, *p* < 0.01, Cohen’s *d* = 0.99, day 3, *t*(11) = −6.95, *p* < 0.001, Cohen’s *d* = 2.01, day 4, *t*(11) = −2.85, *p* < 0.05, Cohen’s *d* = 0.82, day 5, *t*(11) = −3.12, *p* < 0.01, Cohen’s *d* = 0.91). Similar results were observed with the Digital Embryos (day 1, *t*(21) = −2.58, *p* < 0.05, Cohen’s *d* = 0.55, day 2, *t*(21) = −2.91, *p* < 0.01, Cohen’s *d* = 0.62, day 3, *t*(21) = −2.60, *p* < 0.05, Cohen’s *d* = 0.55, day 4, *t*(21) = −4.14, *p* < 0.001, Cohen’s *d* = 0.88, day 5, *t*(21) = −3.55, *p* < 0.01, Cohen’s *d* = 0.76).

Furthermore, the BF_10_ revealed that the overall preference score is 41204 times more likely than the null hypothesis. Following Lee and Wagenmakers classification^69^, the BF_10_ provides extreme evidence that the chicks possess a strong preference for the fast-rotating stimuli when displayed at a low frame rate (24 fps).

Both chicks tested with the cube and with the Digital Embryo showed a preference for the fast-rotating stimulus. On day 1, the preference was more robust in the group exposed to the cube than in the group exposed to the Digital Embryos. On day 1, chicks tested with the rotating cubes spent 70% (+/− 3.41 SEM) of their time close to the fast-rotating object, while chicks tested with the rotating Digital Embryos spent 56% (+/− 2.20 SEM) of their time close to the fast-rotating object. Moreover, the preference toward the fast-rotating cube decreased with time until it reached 59% (+/− 2.95 SEM) on day 5. In contrast, the preference toward the fast-rotating Digital Embryos increased with time until it reached 64% (+/− 3.80 SEM) on day 5.

## General Discussion

This study aimed to re-investigate the spontaneous preference for slow- (in comparison to fast-) rotating objects described in a previous study^45^. Firstly, I tried to duplicate the preference for slow-moving objects using a simple blue cube. Secondly, I investigated whether the preference was affected by the colour and shape of the stimulus using differents, more complex stimuli. Thirdly, I investigated how the preference was influenced by the frame frequency of the videos displaying the stimuli. In all three experiments, I was unable to find a preference for slowly rotating objects. Instead I have found a preference for quickly rotating objects.

The preference I observed agrees well with previous literature on filial imprinting. Soon after hatching, chicks are sensitive for a stimulus to imprint on. As mentioned in the introduction, they possess many predispositions to help them narrow their choice toward specific cues. Among all the predispositions described concerning motion, chicks have a general preference for movements associated with animate stimuli^34,39–41,43,44,73^. The results may fit into this pattern of preference since fast-moving objects are more likely to be considered as animate than slow-moving objects^74^. Classical imprinting literature from the 1960s has also reported that the faster an object moves (or flicker), the more attractive it seems to young birds, so that they would approach and imprint on it^46–49^.

In the literature, spontaneous preferences have been described by focusing on the first minutes and first choices after exposure to some stimuli^37^. In such experimental setting, the attraction observed toward a particular stimulus cannot be explained by learning; therefore, the term ‘spontaneous’ is used appropriately. Prolonged exposure paradigms such as used here and in the replicated study^45^ provide a promising alternative to study animal’s predispositions. Nevertheless, using this kind of paradigm, the preference toward a particular stimulus cannot be defined as a spontaneous preference since it is the result of at least two mechanisms influencing one another, predispositions and learning.

Given that the frequency of frames used for stimuli presentation did not affect the direction of the preference for quickly-moving objects in my experiments, it could be that the discrepancy between my results and Wood’s^45^ results is due to the monitor screen themselves, and that the monitors used in Wood’s study might have been below the flicker frequency fusion threshold of birds^71,72^. Further research would be needed to understand this discrepancy. Meanwhile, these results demonstrate the necessity of controlling the temporal frequency of the monitor when displaying moving stimuli to avian species. Differences between strains also need to be considered. Unfortunately, Wood^45^ did not sex his animals, and this could be important because in some strains, male and female chicks show opposite preferences in some tasks^39^. In our strain, however, no sex differences were observed on this particular task.

## Supplementary information

Supplementary information

Supplementary information2

Supplementary information3

Supplementary information4

Supplementary information5

Supplementary information6

## Data Availability

The datasets generated during and/or analysed during the current study are available on figshare, [10.6084/m9.figshare.11637327].
